# Unravelling mesenteric fibrosis in small intestinal neuroendocrine tumours and implications for clinical management

**DOI:** 10.1530/ERC-25-0116

**Published:** 2026-05-15

**Authors:** Maria Castanho Martins, Eva van der Slik, Johannes Hofland, Anela Blažević, Harry Hodgetts, Dalvinder Mandair, Christos Toumpanakis, Martyn E Caplin, Leo J Hofland, Wouter W de Herder, Krista Rombouts, Richard A Feelders

**Affiliations:** ^1^ Regenerative Medicine and Fibrosis Group, Institute for Liver and Digestive Health, Royal Free Hospital, University College London, London, UK; ^2^ Department of Internal Medicine, Sector Endocrinology, ENETS Center of Excellence, Erasmus MC Cancer Institute, Rotterdam, The Netherlands; ^3^ Neuroendocrine Tumour Unit, ENETS Centre of Excellence, Royal Free London NHS Foundation Trust, London, UK; ^4^ Holman Division of Endocrinology, Diabetes and Metabolism, New York University Langone Medical Center, New York, USA

**Keywords:** narrative review, small intestinal neuroendocrine tumour, mesenteric metastasis, mesenteric fibrosis

## Abstract

Mesenteric fibrosis (MF) is an extensive fibrotic reaction common in small intestine neuroendocrine tumours (SI-NETs) and develops around mesenteric metastasis. While primary SI-NETs can remain asymptomatic for years, mesenteric metastasis (MM) and the resulting MF frequently leads to serious complications. Despite its clear impact on quality of life, MF pathophysiology remains poorly understood and treatment options are currently limited to surgical interventions. This review summarizes the literature on MF in SI-NET patients, emphasizing gaps in knowledge and potential research directions. Clinical research confirms that MF is a frequent complication, often causing severe morbidity, including mesenteric ischaemia and obstruction. Addressing these issues requires further research into MF pathogenesis and contributory factors, such as elastic vascular sclerosis (EVS), which are currently underexplored. Advancing this field will necessitate collaboration, supported by clear definitions and standardized diagnostic and grading systems for MF linked to clinical data, which are presently lacking. While MF-directed translational research remains scarce, recent studies focusing on tumour microenvironment have begun to uncover possible pathways involved in MF. Serotonin, pro-fibrotic growth factors and cytokines may play a role in activating cancer-associated fibroblasts and immune cells within the tumour microenvironment, aggravating extracellular matrix protein deposition and possibly creating positive feedback loops that promote fibrosis. Recently classified as an organ, the mesentery may harbour unique properties for stimulating the establishment of MF. Emerging research is paving the way for new MF treatment options that are urgently needed.

## Introduction

The mesentery has only recently been recognized as a separate organ (1). It is composed of two layers of peritoneum that enclose blood vessels, lymphatic structures and adipose tissue. The peritoneum itself is a thin serous membrane lining the abdominal cavity and covering several abdominal organs. The peritoneal ligaments and mesentery act as boundaries and conduits for disease processes.

One such disease process is mesenteric metastasis (MM) secondary to small intestinal neuroendocrine tumours (SI-NETs). SI-NETs are relatively rare neoplasms originating from neuroendocrine cells that secrete bioactive compounds to regulate both local and distant organ functions (2). The incidence of NETs, including SI-NETs, has increased substantially in the past decades (3, 4). At diagnosis, over 50% of SI-NETs have metastasized to the mesentery (5, 6), underscoring their high metastatic potential despite their typically slow growth. Frequently, dense fibrotic tissue – known as mesenteric fibrosis (MF) – develops around these mesenteric metastases (6). This fibrosis consists of collagen and other connective tissue proteins, with recent proteomic analysis revealing overexpression of certain collagen types, such as COL12A1, in the stroma of patients with MF (7).

Depending on the degree of MF and its location in the mesentery, various severe complications such as ischaemia and bowel obstruction may arise, significantly reducing quality of life (8). The diagnosis and grading of MF are not standardized, leading to variability in its assessment (8). Regarding the pathogenesis, MF is thought to entail serotonin overproduction and growth factors, but the extent of their involvement remains debated due to limited literature (5). Emerging research has highlighted the mesenteric tumour microenvironment, particularly the potential role of cancer-associated fibroblasts (CAFs), in MF progression (5).

Currently, surgery is the only available treatment for MF; however, its effectiveness is often hampered by extensive fibrosis or the central location along the mesenteric root. Encouragingly, recent insights into MF pathogenesis provide promising opportunities for the development of novel therapeutic strategies. This review aims to address these challenges by synthesizing the current literature, advancing our understanding of MF and identifying avenues for future research and treatment development.

## Clinical aspects of mesenteric fibrosis in SI-NET patients

### Epidemiology

MF is commonly observed around MM in patients with SI-NETs (5). While the development of mesenteric metastases and fibrosis appears interconnected, the extent of fibrosis varies significantly between patients. Some may not develop MF at all, although this group might be smaller than previously expected. A recent study (9) revealed that even when a CT scan appears negative for MF, histopathological examination can still detect fibrosis, usually as a limited fibrotic capsule. Since this fibrotic reaction develops almost exclusively around MM, the prevalence of both MM and MF will be briefly reviewed.

Retrospective studies from 1990 to 2021 (6, 10, 11, 12, 13, 14, 15, 16, 17, 18, 19) estimate MM prevalence in SI-NET patients at approximately 75% (range: 48–91%). MF prevalence ranges from 18 to 57%, with an average of 40%. Among patients with MM, MF occurs in about 50% (range: 30–76%). MM exhibits a highly static behaviour, with growth observed in only 13% of cases for MM > 10 mm on CT imaging (20). In patients without MM at diagnosis, 2.6% developed MM over 40 months. Importantly, most patients received somatostatin analogues (SSAs), which may have inhibited growth.

Determining the exact prevalence of MM and MF is challenging due to varying definitions and diagnostic methods. CT imaging may underestimate MF (9), while surgical or histopathological assessments can introduce selection bias. Additionally, many studies do not distinguish mesenteric metastases originating from tumour deposits versus lymph nodes, a point further discussed in the section titled ‘Histopathology of the mesenteric mass’.

### Risk factors for MF development

#### Presence and size of the mesenteric mass

The most critical risk factor for developing MF is the presence of a mesenteric mass or metastatic lymph node in the mesentery (6), with studies suggesting that mass sizes greater than 24 mm (*n* = 81) (19) and 27.5 mm (*n* = 559) (6) may independently increase risk. Accurately sizing a mesenteric mass on CT can be challenging because individual nodes often coalesce into a conglomerate surrounded by MF (21).

Without mesenteric involvement, desmoplastic reactions are exceedingly rare, although isolated cases of fibrosis without a mesenteric mass have been reported (12, 22, 23). These cases include primary tumours breaching into the mesentery, causing a desmoplastic reaction. Additionally, in less than 5% of MF cases, fibrosis develops around small lymph nodes (<10 mm), not meeting the standard definition of a mesenteric mass (6, 17). These findings indicate that the local tumour–mesenteric microenvironment may play a role in driving fibrosis.

#### Sex differences

A male predominance of MM was observed in a cohort of 530 patients (20), with male sex identified as the only significant predictor of MM growth. A subsequent study involving 559 SI-NET patients (24) reported higher rates of MM (71 vs 58%) and MF (46 vs 37%) in men compared to women, along with slightly larger MMs (median: 30 vs 27 mm) and higher 5-HIAA levels (24). Similarly, a retrospective analysis of 849 patients confirmed male sex as a significant predictor of MM, with higher prevalence of MM (57 vs 42%, *P* < 0.001) and MF (49 vs 39%, *P* < 0.01) in men (25). Subgroup analysis in Blažević *et al.* (24) found no significant sex difference in MF development among patients with MM, suggesting male sex is primarily associated with metastatic patterns rather than fibrosis. The protective effect of female sex was most pronounced in women younger than 50 but diminished with age, suggesting a mesentery-specific influence of sex hormones. The role of sex has also been explored in relation to overall survival, with some studies reporting improved outcomes in females – particularly pre-menopausal women (*n* = 73,521) (26) – while others found no association (*n* = 559, *n* = 147, *n* = 14,834) (27, 28, 29).

#### Serotonin levels

Increased serotonin levels, reflected by 5-HIAA, are linked to MM and the development of MF. These systemically measured serotonin levels likely originate from liver metastases rather than local production in the mesentery, as serotonin produced locally is largely metabolized by the liver via the portal system before entering circulation (30). A cohort study of 52 patients found a strong correlation between elevated platelet serotonin levels and MM (15). This was confirmed by a large cohort study (*n* = 559) (6) that identified 24 h urinary 5-HIAA as a risk factor for mesenteric masses. Regarding serotonin and MF, a study of 81 SI-NET patients (19) demonstrated that urinary 5-HIAA levels ≥395 μmol/day increased the likelihood of MF, a finding supported by two large cohort studies (*n* = 559 and *n* = 387) (6, 18) that confirmed a significant association between urinary 5-HIAA excretion and MF. Interestingly, a cohort of 147 patients with MM and MF (28) reported no significant correlation between radiological MF grades (mild to severe) and urinary 5-HIAA levels, in the absence of a control group without MF.

#### Other factors: age, grade and CgA level

Age > 55.8 years was identified as a risk factor for the development of MM in one study, but it was not associated with MF in a cohort of 559 patients (6). Additionally, no association was found between MM and MF regarding tumour grade, Ki-67 expression (19, 24) or chromogranin A (CgA) levels (24).

### Symptoms and complications caused by mesenteric masses and MF

Most SI-NETs are diagnosed at a later stage, after symptoms have already manifested and persisted for a long duration before diagnosis (14, 18). Retrospective cohort studies indicate that 76–84% of patients were symptomatic at diagnosis: 36–51% experienced gastrointestinal (GI) complaints, 11–16% presented with carcinoid syndrome (CS), and 22–24% exhibited both CS and GI symptoms (14, 18). Among symptomatic SI-NET patients, the majority presented with abdominal pain (31). Intermittent small intestinal obstruction was common, while others experienced nonspecific abdominal pain. Over time, symptoms such as diarrhoea, malnutrition and weight loss become more prominent, along with clinical signs such as abdominal masses and GI bleeding (14, 18).

Studies highlight the crucial role of MM and MF in causing symptoms in SI-NET patients. In 1961, Moertel *et al.* observed that non-metastasized SI-NETs were often incidental findings, while 93% of symptomatic patients had metastatic lesions (versus 9% in incidental findings), suggesting that complications are more often caused by metastases, including MM. Removal of mesenteric lymph node metastases significantly alleviated symptoms, including diarrhoea and abdominal pain (10). Among patients with MF, 62–73% were symptomatic, experiencing intermittent bowel obstruction or postprandial pain (32, 33). In a surgical series, 80% of SI-NET patients requiring laparotomy had marked MF (33).

#### Ischaemia

The association between SI-NETs and mesenteric ischaemia is well documented, especially in patients with MF (34, 35, 36). Furthermore, a large retrospective cohort study (*n* = 824) demonstrated that mesenteric ischaemia is an independent prognostic factor for survival in stage IV disease (37). Reported prevalence estimates for mesenteric ischaemia or venous stasis range from 5 to 15%, with higher rates observed in patients with more pronounced MF (6, 11, 17, 18, 19, 28, 31, 37).

**Figure 1:**
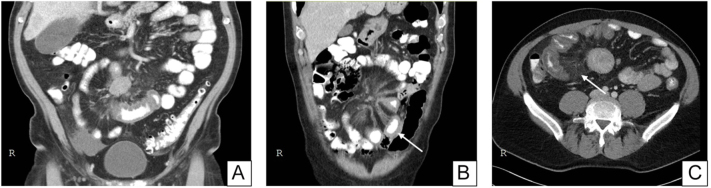
CT images of mesenteric fibrosis in SI-NET patients. (A and B) Coronal CT images with the characteristic ‘spokeswheel’ pattern of mesenteric fibrosis (MF), showing radiating soft tissue strands originating from a mesenteric metastasis. In panel (B), the white arrow highlights an oedematous, thickened intestinal wall caused by venous stasis, which may lead to mesenteric ischaemia. Additionally, on an axial CT image, MF is depicted in panel C as a ‘misty mesentery’, characterized by increased attenuation of the fat tissue. A full colour version of this figure is available at https://doi.org/10.1530/ERC-25-0116.

Ischaemia and impaired intestinal venous drainage can present with symptoms such as postprandial abdominal pain, severe weight loss, malabsorption, frequent diarrhoea and ascites (38, 39, 40). CT imaging signs of venous stasis include intestinal wall thickening (see Fig. 1B), tortuous mesenteric collateral veins, obstructed venous blood flow through the superior mesenteric vein (SMV) and associated findings such as ascites (40).

Mesenteric ischaemia can be attributed to compression of mesenteric vessels by MM and MF, leading to venous stasis and potentially progressing to venous gangrene. In a minority of patients, arterial circulation is also compromised (38). Another possible pathophysiological mechanism is elastic vascular sclerosis (EVS), characterized by elastic tissue proliferation within the adventitia and intima of mesenteric arteries and veins (35, 36), further discussed in the section titled ‘Elastic vascular sclerosis (EVS)’.

#### Obstruction

Bowel obstruction is a common complication of SI-NETs, particularly due to the presence of MM and associated MF. Patients may present with acute intestinal obstruction or episodes of obstruction with intermittent colicky pain (22). A large cohort study (*n* = 559) reported a history of mechanical ileus in 23.3% of patients with MF versus 14.4% without MF (6).

**Figure 2:**
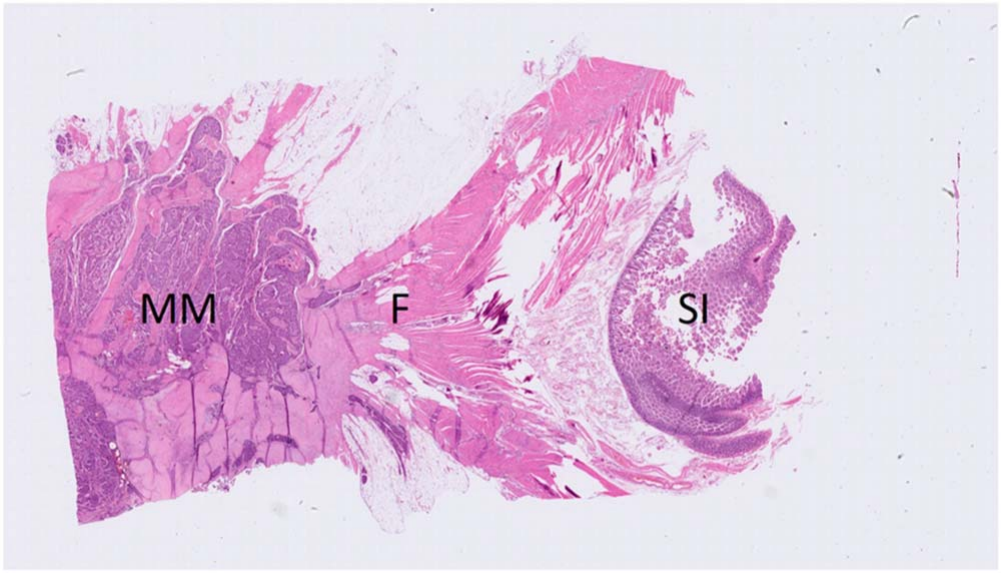
Haematoxylin–eosin (HE) slide of a mesenteric metastasis and surrounding mesenteric fibrosis. This HE-stained slide shows a surgically resected mesenteric metastasis along with the adjacent intestinal segment from a SI-NET patient. On the left, the mesenteric metastasis (MM) is visualized, with thick fibrotic strands (F) radiating outwards into the mesentery, extending towards the bowel wall of the adjacent intestinal segment on the right (SI). This fibrotic traction may result in (partial) bowel obstruction. A full colour version of this figure is available at https://doi.org/10.1530/ERC-25-0116.

Pathophysiologically, retraction towards the root of the mesentery by MM and associated MF can lead to shrinkage and fixation to the retroperitoneum, causing angulation and kinking of bowel loops and ultimately obstruction (41). MF can also exert traction directly on the bowel wall (see Fig. 2). Extensive MF encasing the bowel, combined with hypertrophic muscular thickening, may contribute to diffuse luminal narrowing. Additionally, large MMs can result in volvulus. A small case series of 37 patients reported that, in cases of obstruction, this was mainly attributed to the presence of MM and/or MF, while only 33% were caused by the primary tumour, such as through intussusception (22, 31).

#### Effect on resection and overall survival

Several studies have explored the impact of MF on surgical outcomes. In one cohort, 28% of mesenteric masses were deemed unresectable due to severe desmoplasia (*n* = 37) (11). Another study reported residual MM in 63, 100 and 88% of cases (387 patients) following emergency surgery for bowel obstruction, perforation and mesenteric ischaemia, respectively (18). No significant difference in overall survival (OS) was found between R0, R1 and R2 resections. Completeness of resection may, therefore, have limited influence on outcomes in metastatic disease. Furthermore, among 36 patients undergoing laparotomy for SI-NET, the absence of extensive fibrosis was associated with successful primary tumour and MM resection (42). Notably, surgical specimens were significantly longer in patients with MF.

Different studies have highlighted the association between MM (with or without MF) and OS, accounting for variables such as tumour stage, age, metastases, CgA, 5-HIAA level, tumour grade and sex (18, 28). In one cohort of 70 SI-NET patients, the number of mesenteric tumour deposits (MTDs) was significantly associated with disease-specific survival (DSS), rather than lymph node metastases or MM size (*n* = 70) (43). Another study identified MM > 2 cm as a prognostic factor for OS (*n* = 106) (13).

The relationship between MF and OS remains ambiguous but is gradually becoming clearer. Laskaratos *et al.* observed no significant association between OS and MF severity (mild, moderate or severe) in a cohort of 147 patients (28), although the severe fibrosis subgroup (*n* = 15) might lack statistical power. Blažević *et al.* (6) found that MF was associated with worse OS (median: 102 vs 174 months, *n* = 559) in univariate analysis, although multivariate models identified age, tumour grade, CgA, liver metastases and 5-HIAA levels as stronger predictors of OS. Symptomatic versus asymptomatic MF status was not significantly associated with OS in two cohort studies (33, 44).

These findings suggest that age, tumour aggressiveness and disease burden primarily dictate survival outcomes. Nonetheless, emerging evidence indicates that MF may not merely reflect advanced disease but actively contribute to its progression. Bosch *et al.* (2018) and van der Slik *et al.* (2025) demonstrated that patients with MF more frequently exhibited distant metastases (23, 45). Furthermore, extramural vascular invasion – an increasingly recognized risk factor for metastasis and poor prognosis – was strongly associated with MF presence (*κ* = 0.85; 95% CI: (0.54, 1.00)) (46, 47). It is hypothesized that MF encasing blood vessels may predispose them to invasion. The strong correlation between MF and tumour stage might potentially obscure MF’s independent prognostic value. Notably, in a regression analysis excluding tumour stage and including age, sex and tumour size, MF emerged as a significant predictor of survival (*n* = 43), supporting its potential role in disease progression (46). The relevance of stromal remodelling is underscored by the recent identification of four molecular subtypes in SI-NETs (*n* = 122), where a mesenchymal subtype characterized by CAF infiltration in the primary tumour was associated with worse prognosis (48).

### Other fibrotic complications in SI-NET patients in relation to MF

#### Carcinoid heart disease (CHD)

CHD has been reported in at least 20% of patients with CS (49) and predominantly involves valvular fibrosis, mainly affecting the right-sided heart valves, especially the tricuspid valve. Left-sided valvular involvement is rare but can occur in patients with an inter-atrial shunt or prolonged elevated 5-HIAA levels (50).

Serotonin overproduction is thought to play a key role in both CHD and MF. However, evidence linking serotonin to CHD is more robust (18, 51, 52, 53, 54). For instance, in CHD, the 5-HT2B receptor is highly expressed in heart valves, while its expression in MM remains unexplored (53, 54). Furthermore, serotonin’s role in MF development appears less significant, as Laskaratos *et al.* (18) demonstrated a stronger correlation between 5-HIAA levels and CHD than MF in a cohort of 387 patients.

In addition, for both CHD and MF, findings indicate that pathophysiological mechanisms beyond circulating serotonin likely contribute (52, 55, 56). However, at least partially distinct underlying mechanisms are implied by studies reporting no significant association between CHD and MF (387 patients) (12, 18). In a cohort of 200 SI-NET patients with CHD only 10% showed signs of MF, compared to 15% in patients without CHD, further supporting the lack of association (57).

#### Retroperitoneal fibrosis

A cohort study of 31 patients reported a 6% incidence of retroperitoneal fibrosis on CT imaging (12), and several case reports describe the association between retroperitoneal fibrosis and SI-NETs, which can co-occur with elevated serotonin production and carcinoid syndrome (CS) (58). However, some patients present without CS (59) or solely with a primary ileal tumour (60). Severe retroperitoneal fibrosis can lead to ureteric obstruction, hydronephrosis and ultimately kidney failure (61, 62). Analogous to MF, local serotonin synthesis may contribute to pathogenesis, as substantiated by elevated concentrations of serotonin and its metabolites observed in the peritoneal fluid (63, 64). Consistent with this, some patients were successfully treated with combination therapies including octreotide (65, 66).

#### Elastic vascular sclerosis (EVS)

Elastic vascular sclerosis (EVS) is a less well-known entity associated with SI-NETs, characterized by the proliferation of intimal, adventitial and perivascular elastic tissue affecting both mesenteric arteries and veins (35). EVS is distinct from MF and CHD due to its lack of collagen and adventitial location. In 25 SI-NET cases (35), small vessels near the primary tumour were mostly unaffected, but EVS appeared in 85% of distant mesenteric vessels, often concealed by MF, retraction or kinking by local tumour spread. EVS was absent in NETs from other primary sites.

An important role of EVS in mesenteric ischaemia is suggested since four of the five ischaemic cases showed EVS-related lumen narrowing. EVS is not a specific indicator, however, as it is regularly not accompanied by mesenteric ischaemia (67, 68). Although, in all ischaemic cases, large metastatic deposits were present in the mesentery, these deposits did not narrow or occlude the vessel lumen while EVS did. In addition, all ischaemic cases displayed MF. Intestinal ischaemia might therefore result from a combination of MF and EVS as these conditions quite often co-occur but also have shown to be able to cause intestinal ischaemia in isolation (58, 69).

EVS pathogenesis may involve mechanical forces, elastogenic factors released by MM surrounding the affected vessels or direct tumour interactions with vascular fibroblasts or smooth muscle cells (68). Elevated bone morphogenetic protein 4 expression in EVS-associated tumours, independent of the presence of MF, might suggest tumour-associated vascular remodelling (70).

#### Cutaneous scleroderma-like lesions

Several case reports have documented an association between scleroderma-like lesions and SI-NETs (71, 72, 73). Durward *et al.* (74) reviewed seven cases, concluding that all patients had SI-NETs with liver involvement and subsequently developed CHD. Notably, all patients first presented with scleroderma-like lesions on their legs, but none exhibited clinical features or autoantibodies indicative of systemic sclerosis. These findings suggest that the association between CS and certain scleroderma-like features is likely more than coincidental. Supporting this, a recent case study reported successful treatment of CS-related scleroderma-like lesions with octreotide (75).

In conclusion, several other fibrotic conditions may develop in patients with SI-NETs apart from MF. Their occurrence at sites distant from the tumour suggests a role for circulating pro-fibrotic factors. Serotonin is often proposed as a contributor, although its involvement is not consistently demonstrated, and mechanisms beyond serotonin likely contribute to some manifestations. Why fibrosis arises at these specific sites, and only in a subset of patients, is not yet fully understood and might differ between fibrotic conditions.

## Diagnosing and grading mesenteric fibrosis

Significant variability exists in MF assessment across studies, which may cause discrepant outcomes. The main approaches – histopathological and radiological – will be outlined below together with emerging developments in the field.

### Histopathology

#### Histopathology of the mesenteric mass

The mesenteric mass is generally thought to originate in lymph nodes, although histologic confirmation is limited – residual nodal tissue was found in only 7 of 21 cases (76). In the remaining cases, the lymph node may have been fully replaced, or the mesenteric mass could have arisen from angiogenic or peritoneal spread. Mesenteric tumour deposits (MTDs) are associated with poorer disease-specific survival (*n* = 70) (43) and often mimic nodal disease but typically show irregular contours, perineural invasion and thick-walled vessel entrapment. Occasionally, they exhibit rounded contours and peripheral inflammatory infiltrates, resembling lymph nodes (77). Differentiation remains challenging, with moderate interobserver agreement in colorectal cancer (*κ* = 0.48) and better concordance in SI-NETs (*κ* = 0.64) (78). Preferred criteria include irregular shape and nerve/vessel entrapment for MTDs and round shape with peripheral lymphocytes for lymph nodes. However, this distinction remains under development and still lacks universal application in SI-NET patients.

#### Histopathology of MF

The most widely used grading system for mesenteric fibrosis, described by Pantongrag-Brown *et al.* (76), classifies fibrosis based on fibrous band width: grade 1 (<1 mm), grade 2 (0.5–2 mm) and grade 3 (>2 mm). These grades align with CT imaging findings but have not been correlated with clinical data. Additionally, the specific location of measurement (e.g. inclusion of the capsule or only intratumoural tissue) remains undefined. A recent study incorporated an additional parameter: the collagen proportionate area (CPA) using digital assessment of collagen measurement on collagen-stained slides, representing the percentage of collagen in the tumour stroma (9). This quantitative method, validated in liver cirrhosis (79, 80), showed a stronger correlation with radiological scoring systems to assess MF. Moreover, recent data indicate that these CPA values, specifically those reflecting intratumoural tissue while excluding the fibrotic capsule, correlate well with clinical parameters such as tumour stage (III or IV) and the presence of abdominal pain – unlike other histopathological and radiological metrics (Pantongrag-Brown *et al.* (76), Van der Slik *et al.* (23)). Additionally, other approaches have been utilized; for example, extensive fibrosis has been defined as stromal fibrosis exceeding 50% of the tumour size relative to the neoplastic cellular component (70). In conclusion, a more standardized fibrosis scoring system, validated with clinical data, is highly desired to enhance consistency and applicability.

### Radiology

MM and surrounding MF can lead to foreshortening, thickening and kinking of the mesentery. This creates a pathognomonic pattern for mesenteric involvement, including contraction of the mesenteric branches of the superior mesenteric artery towards the root of the mesentery, causing bowel loops to appear arranged in a stellate or spoke-like pattern or many small punctate densities, while the vasa recta to the small bowel remain normal (see Fig. 1A and B) (41, 81, 82, 83). Pantongrag-Brown *et al.* (76) were the first to correlate radiological findings with histopathological scoring. CT stranding was classified as mild (<10 thin strands), moderate (>10 thin or <10 thick strands) or marked (>10 thick strands), while MF on HE-stained slides was graded by fibrous band width. Radiological stranding generally reflected fibrosis severity, with moderate-to-marked strands more common in high-grade cases.

In addition to the typical stellate pattern, hyperdense linear images resembling the comb sign (84) and increased mesenteric attenuation can also suggest MF (see Fig. 1C) (85), although these findings are less specific than the stellate pattern.

Although most studies use the radiological scoring system described by Pantongrag-Brown *et al.* (76), it may underdiagnose MF. Laskaratos *et al.* (2020) reported that up to 40% of histologically confirmed fibrosis was not detected on imaging or intraoperatively. To improve sensitivity, several studies have incorporated additional radiological features – such as misty mesenteric attenuation – into their definitions of MF (12, 15).

### New developments

#### NETest and fibrosome

The NETest is a PCR-based 51-transcript signature with good sensitivity and specificity for diagnosing gastroenteropancreatic NETs. It outperforms conventional secretory biomarkers and correlates with progression-free survival (PFS) and overall survival (OS) in patients with metastatic SI-NENs (86, 87, 88). A subset of five genes from the NETest (APLP2, BNIP3L, CTGF, CD59 and FDZ7), collectively known as the fibrosome, were tested in pre-operative blood samples of a cohort of 20 SI-NET patients without CHD or other fibrotic disorders. The fibrosome accurately predicted the presence of both microscopic (image-negative) and macroscopic fibrosis (9). In another study, the protein expression levels of four of these five fibrosis-related markers (APLP2, BNIP3L, CD59 and DKK3) were investigated immunohistologically in primary tumours of 128 SI-NETs (89). Protein expression was assessed in relation to MF, but no significant associations were observed. Importantly, liquid biopsy gene expression (9) was compared to primary tumour protein expression (89), which may not reflect heterogeneous expression in the primary tumour, mesenteric metastases and the stromal microenvironment.

In conclusion, the fibrosome shows promise as a diagnostic tool for MF. However, larger multicentre studies are needed to validate its sensitivity and specificity for carcinoid-related fibrosis, assess performance in other fibrotic conditions and determine whether it can accurately grade MF severity.

#### Radiomics

Blazevic *et al.* (90) developed a predictive radiomics model to identify patients prone to developing complications from mesenteric metastasis and fibrosis, who may benefit from prophylactic surgery. Among all radiomics features tested, only the surrounding mesentery (SM) significantly differed between asymptomatic and symptomatic patients (*n* = 68 patients), with 93% based on texture features. While systemic evaluation by clinicians scored equally well, the advantage of this model is its independence from the observer, requiring no personal training or experience.

#### FAPI-based PET/CT imaging

The detection of mesenteric masses has significantly improved with functional imaging using ^68^Ga-DOTATOC PET/CT compared to conventional CT (91, 92). However, visualizing fibrosis remains challenging with current imaging techniques. Fibroblast activation protein (FAP) is overexpressed on cancer-associated fibroblasts (CAFs) across various tumour types (93). A promising development in oncologic imaging is the use of FAPI ligands or FAP inhibitors as PET/CT radiotracers, which in several cases have demonstrated improved diagnostic accuracy (94). Additionally, FAPI-based PET/CT has shown efficacy in detecting fibrotic tissue in several conditions (93, 95, 96, 97, 98). Therefore, FAPI-based PET/CT imaging may represent a promising tool for diagnosing and grading SI-NET-associated MF, although no studies have yet evaluated this application.

## Pathogenesis of mesenteric fibrosis

### Tumour microenvironment in MF

The tumour microenvironment (TME) has gained increasing importance in the past decades as a crucial player to understand cancer initiation, progression and metastasis as it harbours an essential role in promoting inflammation and fibrosis, which can further drive cancer development. The TME is a complex disease-specific structure composed of CAFs, immune cells, vessels and the extracellular matrix (ECM). Although SI-NET microenvironment has not extensively been described, the knowledge on MM microenvironment within the context of MF is even less explored.

#### Immune cells

The SI-NET immune tumour microenvironment (TIME) has been described as ‘cold’ by few studies due to lack of immune cell infiltrates and expression of immune markers (99). Immune checkpoint inhibitor therapy has been successful in various cancers; however, clinical trials so far have shown limited efficacy for its use in SI-NETs (100). This may be due to the low expression of PD-L1 in SI-NET primary tumours and metastases (101, 102, 103, 104, 105). PD-L1 expression can be upregulated in fibroblasts by transforming growth factor beta (TGF-β), a master regulator of fibrosis needed for fibroblast activation, and additionally enhance their invasive and migratory potential (106), which could prove relevant in the setting of MF, although no studies have yet evaluated MM stromal/tumoural compartment expression.

Macrophages play a major role in tissue remodelling and fibrosis, and tumour-associated macrophages (TAMs) can also be pro-tumourigenic within the TME (107). Macrophage infiltration has been reported to be higher in metastasis compared to primary tumours of pancreatic NET (pNETs) (108, 109), with high infiltration correlating with disease recurrence and poor disease-free or recurrence-free survival (109, 110, 111). A study by Busse *et al.* showed that low-grade primary ileal NETs had a higher TAM infiltration compared to healthy controls (112), although further studies on TAM mesenteric mass infiltration are lacking.

Other immune cells, such as dendritic cells and neutrophils, which have also been described in NETs (111, 112), can further play a role in fibrosis through the production of pro-inflammatory cytokines and stimulation of fibroblasts (113).

#### Cancer-associated fibroblasts (CAFs)

Fibroblasts are the main cells involved in the development of fibrosis. In the context of cancer, CAFs can aid tumour stroma development and promote tumour growth. For this reason, CAFs are predicted to be a key contributor to MF development in SI-NET patients (114, 115).

CAFs are activated fibroblasts, staining positive for classical activation marker αSMA and usually presenting with more aggressive phenotypes, such as increased proliferation, migration, ECM deposition and secretion of autocrine and paracrine signalling molecules (116). SI-NETs have higher αSMA-positivity in the stroma of primary tumours and metastases when compared to other NETs (114). A study by Graf *et al.* confirmed enrichment of αSMA-positive cells in the stroma of lymph node metastasis of SI-NET patients presenting with MF (*n* = 11) compared to those without (*n* = 13) (117). Isolated CAFs from SI-NETs have also been shown to retain αSMA-positivity when grown *in vitro* (118, 119).

A better understanding of CAF populations within SI-NET MF is needed to understand pathophysiology and develop treatments, as several studies have highlighted that CAF populations can be composed of cell clusters with distinct behaviours and expression profiles (120, 121, 122). Thus, single-cell or spatial multi-omic analysis of MF samples could help elucidate on key MF characteristics.

#### Extracellular matrix (ECM)

The ECM is the final TME component essential in fibrosis. The ECM is composed of several fibrillar and non-fibrillar proteins, which can regulate cell function and behaviour through mechanical and biochemical cues (123). Fibroblasts produce ECM components and maintain this structure. During disease, dysregulated fibroblasts cause disorganized fibril arrangement and increased stiffness, which will act on fibroblasts and maintain their activated state, forming a positive feedback loop (123). SI enterochromaffin cells are mechanosensitive, and serotonin release can be modulated by mechanical forces (124). Piezo2, a mechanosensitive ion channel, has been shown to be selectively expressed by these cells (125, 126). It is yet to be confirmed if SI-NET cancer cells retain mechanosensitivity and if fibrotic-induced tissue stiffness triggers higher serotonin release, forming another positive feedback loop by acting on fibroblasts.

Blazevic *et al.* have performed proteomics analysis on the tumour and stroma compartments of primary tumours and paired MM tissues of patients with (*n* = 6) and without MF (*n* = 6) (127, 128). They reported that 10 proteins involved in metabolizing serotonin were significantly decreased in the stromal compartment of MF-positive MM samples compared to MF-negative samples, while this difference was not seen for the MM tumour compartment nor for the primary tumours (127). Investigators also reported the enrichment of matrix proteins in MF-positive MM stroma, including SRPX, ASPN, HTRA1, PCOLCE and KRT80, as well as an enrichment of different collagen molecules (128). More research is needed to further characterize the ECM compartment separately from the cellular compartments, in both MF and non-MF patients, as this could help elucidate which proteins might be responsible for stimulating CAFs and driving sustained fibrosis in the setting of MF.

### Circulating factors

#### Serotonin

Intestinal enterochromaffin cells are one of the major sites of serotonin production outside of the brain. Serotonin is stored in platelets and can be released upon wound healing which, if not resolved, can perpetuate inflammation and potentially progress to chronic wound healing and fibrosis (129). Serotonin has been shown to induce pro-fibrogenic marker expression in healthy and scleroderma-derived fibroblasts in a dose-dependent manner through 5HT-2B stimulation, an effect abolished by TGF-β1 neutralization. Serotonin-stimulating effects on fibroblasts have also been described in liver (130) and lung fibrosis (131, 132). Blazevic *et al.* showed that serotonin metabolism is dysregulated in MF-positive MM stroma (127). Hodgetts *et al.* showed that telotristat ethyl (TE) treatment resulted in significantly decreased proliferation and serotonin secretion in GOT1 cells, with TE further dysregulating pro-fibrotic crosstalk of SI-NET cancer cells and fibroblasts (133). Svejda *et al.* showed that a 5HT-2B inhibitor (PRX-08066) reduced pro-fibrotic marker expression in KRJ-I cells and indirectly in HEK293 cells within a co-culture model (134). However, neither KRJ-I cells nor HEK293 are good representations of their intended cell model – SI-NET cells and fibroblasts, respectively. Further studies are needed to characterize the full role of serotonin in MF and its interaction with other pro-fibrotic factors in order to identify therapeutic windows.

#### Pro-fibrotic and pro-inflammatory factors

Most studies investigating the role pro-fibrotic factors on NETs have either been of clinical nature and not greatly expanded on the molecular mechanism or have used *in vitro* models not representative of SI-NET.

Transforming growth factor beta is a multifunctional molecule with multiple roles in health and disease, which can trigger several signalling pathways that may contribute to fibrosis and cancer, including SMAD, RAS/RAF, TRAF4/6, Rho/ROCK and PI3K/Akt/mTOR (135).

TGF-β isoforms 1, 2 and 3 have pro-fibrogenic activity, and all are expressed at varying levels in SI-NET tissue, as well as TGF-β receptors 1 and 2 (136, 137). A study by Laskaratos *et al.* showed that in patients with MF (*n* = 31), TGF-β mRNA expression was significantly upregulated in MM compared to both primary tumour and normal intestine, while the primary tumour also had significant increased expression compared to the normal tissue, which was not confirmed in patients without MF (*n* = 3) (138). TGF-β-targeting agents have been widely studied for cancer treatment, and some agents have shown acceptable safety and tolerability profiles in clinical trials for solid tumours (139). However, due to the critical role of TGF-β in homeostasis of various tissues, considerable side effects still hinder the application of such therapies (139, 140).

Connective tissue growth factor (CTGF) is an ECM regulatory protein with increased expression in several fibrotic diseases, as well as cancers, including NETs, and can play an important role in establishing metastasis through ECM modulation (141). Furthermore, its effects can be amplified by TGF-β. In SI-NETs, CTGF is expressed by the majority of tumour cells and by activated fibroblasts in tumour stroma, particularly in patients presenting with fibrosis (142, 143). Furthermore, primary fibroblasts isolated from fibrotic SI-NET tumours have significantly increased levels of CTGF gene expression upon TGF-β stimulation (142). Furthermore, circulating CTGF mRNA expression could independently predict the presence of MF in SI-NET patients (9). Despite its relevance for multiple fibrotic conditions, no CTGF-targeting therapies are currently available (140, 144, 145, 146).

Platelet-derived growth factors (PDGFs) are a group of five isoform proteins with mainly paracrine effects, which can activate several downstream pathways, such as PI3K, JNK, MAPK and calcium release (147). PDGFs play a central role in fibrosis development, as described in multiple diseases (e.g. cancer-associated fibrosis; liver, kidney and cardiac fibrosis) (147). PDGF receptor alpha (PDGFRα) are primarily expressed in cancer cells, while PDGFR receptor beta (PDGFRβ) is more commonly expressed in fibroblasts, although both receptors can exert activating and mitogenic functions (148). PDGFRβ is expressed in SI-NET tumour stroma but not tumour cells, with stronger immunoreactivity in metastasis compared to paired primary tumours, while a majority of both tumour and stroma compartments express PDGFRα and PDGF (149, 150, 151). A positive correlation between PDGFRβ staining and Leu5-M staining (a marker for monocytes or macrophages) was also found for SI-NETs but not seen in pancreatic NETs or non-NET samples (149). A tyrosine kinase inhibitor, nintedanib, which targets PDGF, fibroblast growth factor and vascular endothelial growth factor receptors, is currently used in clinic for idiopathic pulmonary fibrosis (IPF) patients (146), although no PDGF-targeting pharmacological intervention has yet been explored for MF.

Besides these classical pro-fibrotic factors, inflammatory cytokines can also contribute towards fibrosis development. Bowden *et al.* described increased IL-6 secretion associated with carcinoid-related fibroblasts (152). Increased IL-6 serum levels have also been described in SI-NETs compared to healthy controls; however, the presence and correlation with MM or MF was not analysed (153). IL-6 immunohistochemical expression has further been reported in the peritumoural stroma of intestinal NETs by endothelial cells, fibroblasts and immune cells (154). IL-6 can induce macrophage activation in fibrosis, as well as fibroblast activation, promoting secretion of ECM proteins (107), thus being relevant for MF pathogenesis. Tocilizumab, an IL-6-neutralizing antibody, has been tested for systemic sclerosis, which causes tissue fibrosis and inflammation, showing a significant improvement in forced vital capacity in treated patients of a phase III trial despite not meeting trial endpoints (155, 156), thus showing potential for fibrosis therapy.

CCL24, a chemokine associated with M2 macrophage activation, was shown to be upregulated in one of the SI-NET-derived TAM populations when performing single-cell RNA-seq (101). CCL24 has been shown to promote fibrosis in different organs, including the liver, skin and lungs, and its expression can be stimulated by stiffer matrixes (157, 158, 159).

### Signalling pathways

Integrins are cell surface receptors that can interact with the ECM and trigger intracellular signalling regulating cell behaviour. A study by Laskaratos *et al.* analysed gene expression on SI-NET patient tissue samples, highlighting a significant upregulation of *ITGAV* and *ITGAX* in MM of MF-positive SI-NET patients (*n* = 31), with no significant differences seen in tissue from patients without MF (*n* = 3) (138). Integrins have been extensively evaluated for clinical targeting, with several compounds in clinical trials targeting multiple integrin types for application in fibrotic-related diseases, including sclerosing cholangitis, IPF and keloids (160). Alpha V integrin, in particular, has been shown to contribute to fibrosis in several organs, but no specific inhibitors have yet been approved (161).

Hedgehog signalling is often involved in embryonic and tissue homeostasis and has been noted to be active in SI-NETs and fibrotic conditions. When investigating IHC of a series of 33 primary SI-NETs and liver metastasis, 27 were positive for sonic hedgehog and 22 for its downstream target Snail1 (162). Several drugs targeting hedgehog signalling have been approved for cancer treatment, and many are currently in clinical trial phase (163).

Notch signalling plays a role in carcinogenesis and fibrosis, interacting with TGF-β, and has been investigated in SI-NETs pathogenesis. In a recent study by Hodgetts *et al.*, however, no significant changes in mRNA expression were seen for NOTCH1 in tissues from normal intestine, primary tumour and mesenteric metastasis from patients with mesenteric fibrosis (*n* = 31) (133).

The Wnt/beta-catenin signalling pathway is involved in cell proliferation and migration and has been highly implicated in cancer development. In SI-NETs, however, IHC analysis has revealed membranous presence only (inactive form) (162). Furthermore, despite protein levels being significantly upregulated in primary tumours compared to normal intestine tissue, no significant changes were noted between the former and mesenteric mass tissue, nor between different levels of MF severity (133).

### Novel factors

Recent studies focusing on MF pathophysiology have highlighted potential new mechanisms and pathways linked to the disease beyond the classical pro-fibrotic and pro-inflammatory factors already discussed.

As previously mentioned, a study by Blažević *et al.* performed ingenuity pathway analyses on proteomic data from non-MF and MF tumour and stromal compartments, highlighting higher abundance of collagens in MF stromal samples and confirming higher expression by IHC of C9 and COL12A1 in MF tumour and stroma compared to non-MF tissues (128). Hodgetts *et al.* further confirmed significantly increased COL12A1 mRNA expression in MM compared to healthy intestinal tissue of MF patients (133).

Graf *et al.* performed differential gene expression analysis on MF-positive (*n* = 11) or MF-negative (*n* = 13) lymph node metastasis or stromal compartments, showing upregulation of *COMP* (cartilage oligomeric matrix protein), *COL11A1* (also shown to be upregulated at protein level in Blažević *et al.*’s (128) proteomic study) and *CXCL9* in MF stromal samples (117). COMP differential expression in MF was later confirmed by qPCR and IHC, showing that it could be a potential target in disease development (117).

As introduced in a previous section, sex dimorphism may be a risk factor for MF. The proposed mechanisms include the direct pro- and anti-fibrogenic effects mediated by different sex steroid receptors (5). Particularly, oestradiol can modulate the serotonin pathway by increasing tryptophan hydroxylase-2 and serotonin transporter expression while reducing serotonin 1A receptor and monoamine oxidase A/B expression (164). A recent study by Wang *et al.* investigated the effect of sex hormone treatment on 12 primary cell cultures of GEP-NETs (5 PNETs and 6 SI-NETs), including oestradiol, progesterone and testosterone, but none had significant effects on cell viability when compared to controls (165). Moreover, sex hormones have been reported to have a significant role in various fibrotic diseases; oestrogens play a potential protective role in pre-menopausal women (166). Blažević *et al.* showed that primary SI-NETs had a significantly higher mRNA expression of both AR and ESR1 (encoding androgen and oestrogen 1 receptors, respectively), although samples (*n* = 24) were not discriminated by MF presence nor gender. In NETs, case studies have noted improved fibrosis upon tamoxifen treatment (selective oestrogen receptor modulator), although the single-arm HORMONET study did not note a significant improvement in patients with oestrogen-positive tumours receiving tamoxifen therapy (SI-NET *n* = 6) (167). Tamoxifen combination therapy has shown an improvement in relapse-free survival in idiopathic retroperitoneal fibrosis (168), which could present an opportunity for MF treatment in relevant patient groups.

### What makes the mesentery a perfect site for NET-driven fibrosis?

While SI-NETs metastasize to multiple locations, the explosive desmoplastic reaction characteristic of MM seems to be unique to that site. Although fibrosis is a multi-factor reaction with several mechanisms involved, especially in the context of cancer-associated fibrosis, it is likely that the mesentery microenvironment also plays a role in this development.

Descriptions and anatomical representations of the mesentery date as far back as the 16th century with drawings by Leonardo da Vinci. However, it is only recently that the mesentery has been widely accepted as an organ and comprehensive knowledge of its functions is still lacking (169, 170).

The mesentery is able to support organ development, including heterotopic organs, as well as ectopic pregnancies or teratomas (171). Moreover, the mesentery also harbours adipose stem cells, which can drive inflammatory processes in adjacent tissues. In Crohn’s disease, anatomical expansion of the mesentery due to extensive inflammation (172) can trigger adipocyte and fibroblast stimulation, which in turn release cytokines that facilitate sustained inflammation and progression to fibrosis (173). Circulating fibrocytes have also been described in mesenteric inflammatory pathologies with association with inflammation and fibrosis (172). Although not all features may be shared with MF, knowledge from mesenteric pathologies may shed a light on MF pathophysiology.

## Treatment of mesenteric metastasis and related mesenteric fibrosis

### Surgery

For patients with MM/MF presenting with localized and resectable disease, locoregional resective surgery remains the primary treatment option. Surgical radicality must be balanced against the need to preserve bowel function, typically achieved through a mesenteric-sparing technique. Regional mesenteric lymphadenectomy combined with primary tumour resection has been associated with improved survival in patients with SI-NETs (*n* = 1,364) (174). Similarly, for MM/MF patients with locally advanced or distant-stage disease who present with local tumour-related symptoms, surgery or less invasive interventions are considered first-line palliative treatment options (8, 175). Surgery has been shown to alleviate symptoms, particularly through removal of MM, which also significantly improved OS compared with cases where mesenteric metastases persisted (*n* = 314) (10). Survival analyses indicated that patients experiencing symptom relief post-surgery had a better median survival than those with persistent symptoms. In a series of 212 patients, symptom alleviation was reported in 82% of those who underwent surgery, with longer-lasting improvements observed after elective surgery (16). Additionally, Kaplan–Meier analysis showed improved survival for patients undergoing surgery for MF-related complications (*n* = 62) (33).

Controversy persists regarding the role of prophylactic palliative surgery in asymptomatic patients with stage IV disease (176). A recent study leveraging contrasting institutional treatment strategies identified primary tumour resection as an independent predictor of reduced disease-specific mortality (*n* = 145) (177). However, other cohort studies reported no survival benefit from prophylactic surgery (*n* = 559 and *n* = 139) (6, 178). Recent findings (*n* = 387) (18) indicate that OS improves for symptomatic patients undergoing primary tumour and MM resections, but not for asymptomatic patients. In a subgroup analysis in patients with MF, no improvement in OS was observed in the multivariate analysis following primary tumour resection (28). Due to the complexity of the issue, we refer to a recent review by Hallet *et al.* (179), which concluded that resection of the primary/regional tumour for stage IV SI-NETs should be considered to avoid future complications and potentially improve survival, although direct causality is not demonstrated.

#### Resectability

Determining the resectability of primary tumours and mesenteric masses can be challenging. Mesenteric involvement is classified into three subtypes: type A, characterized by resectable disease sparing the mesenteric root; type B, involving nodal metastases and fibrosis adjacent to the SMA and SMV; and type C, characterized by locally advanced or unresectable disease with tumour deposits and fibrosis encasing the SMA and SMV. Successful resection of both primary tumours and mesenteric disease is frequently achievable in types A and B. However, SMA involvement, infiltration of the SMV proximal to the middle colic vein (type C) and signs of MF on CT imaging predict operative failure in SI-NET patients (180, 181). When surgery is not feasible, surgical bypass should be considered for patients with a discrete and symptomatic point of obstruction (182).

### Somatostatin analogues (SSAs)/telotristat

Both telotristat and SSAs are used in the treatment of SI-NETs and inhibit serotonin production, as reflected by reductions in 5-HIAA levels. However, a complete biochemical response is rarely achieved (183, 184). Given the established link between serotonin and MF, and recent evidence that telotristat disrupts SI-NET–fibroblast crosstalk (133), serotonin reduction is hypothesized to inhibit fibrosis formation. Some studies have advocated for higher dosages of SSAs to strive for normalization of 5-HIAA levels (5). Additionally, one study showed that SSAs significantly reduce levels of CTGF and TGF-β (185). SSAs are also known to attenuate fibrosis in animal models of peritoneal, pulmonary and liver fibrosis (186, 187, 188). Despite these findings, neither drug has been studied specifically for the treatment of MF.

### Peptide receptor radionuclide therapy (PRRT)

Blazevic *et al.* (*n* = 68) (20) demonstrated that peptide receptor radionuclide therapy (PRRT) was not effective in reducing the size of MM. An objective response (≥30 reduction in the sum of diameters of all target lesions) was observed in only 12.9%, with a reduction exceeding 30% noted in just 3.8%. Similarly, other studies reported no significant change in the severity of MF following PRRT (*n* = 69) (189) or change in functional volumes (*n* = 20) (190). Interestingly, Mansour *et al.* (*n* = 52) (191) reported that PRRT alleviated MF-related symptoms, with 46% of patients experiencing a clinical response, although no morphologic responses of the MM were noted. PRRT may help stabilize MM, as fewer patients presented with progressive disease after treatment (>33% before PRRT), and MM-related progression-free survival (PFS) was longer compared to non-MM-related PFS.

While PRRT generally has minimal side effects, a possible link to bowel obstruction in patients with peritoneal and mesenteric metastases has been noted (*n* = 159 and *n* = 20) (190, 192). In these studies, 6–15% of patients with mesenteric or peritoneal disease developed bowel obstruction within three months after PRRT, potentially due to an inflammatory response induced by radiation. However, since these patients had advanced mesenteric and/or peritoneal disease, which inherently carries a high risk of obstruction, causality is difficult to establish.

In conclusion, PRRT remains a consideration for MM-associated fibrosis, as it may stabilize tumour growth and relieve symptoms (193). However, clinicians should remain vigilant about the potential, non-negligible risk of bowel obstruction in high-risk patients.

### Potential new treatments

**Figure 3:**
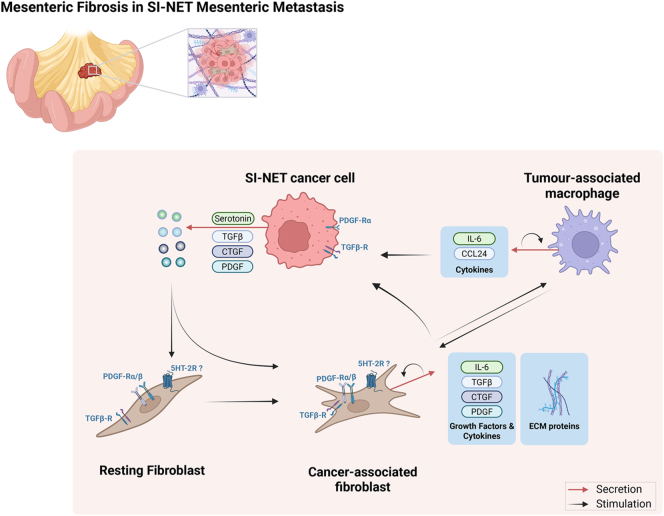
Known and possible mechanisms in mesenteric fibrosis associated with small intestine neuroendocrine tumours. Although the understanding of mesenteric fibrosis (MF) pathophysiology is limited, certain mechanisms of action are known, and recent studies have uncovered potential new pathways. Serotonin, secreted by SI-NET cells, is able to activate and stimulate fibroblasts, converting them into active cancer-associated fibroblasts (CAFs). This activation could contribute to MF, despite the lack of demonstration of 5HT-2B receptor expression on SI-NET fibroblasts. SI-NET cells also secrete TGF-β, CTGF and PDGF, which can all stimulate fibroblast activation and continue to act on CAFs. In turn, CAFs secrete various growth factors and cytokines, as well as secrete ECM proteins, which can have an autocrine effect, as well as act on other cells in the tumour microenvironment, such as tumour-associated macrophages (TAMs) and SI-NET cells. TAMs are also capable of stimulating CAFs via secretion of cytokines, as well as act on the cancer cells. While serotonin secretion can be inhibited by somatostatin analogues, their true effect on attenuating MF or reverting it needs to be further explored and characterized. Targeting growth factors and cytokines may present a new treatment avenue for MF. Created in BioRender. Martins M (2025) https://BioRender.com/5chowes. A full colour version of this figure is available at https://doi.org/10.1530/ERC-25-0116.

Currently, no MF-specific pharmacological treatments are available for SI-NET patients. In the section titled ‘Pathogenesis of mesenteric fibrosis’, we have highlighted potential pathways that could be explored and targeted (Fig. 3), with fibrotic- and inflammation-associated molecules being the broadest approach. Although some novel MF-specific targets have been highlighted in recent studies, the current lack of pharmacological treatments underlines the need for more translational and pre-clinical research in MF with the support of adequate models to better investigate disease mechanisms and potential new targets for treatment.

## Conclusion

This literature review aims to provide a comprehensive overview of mesenteric fibrosis in SI-NET patients, focusing on unresolved research areas and promising developments in the field. The available literature highlights that MF is a frequent complication with significant morbidity, substantially impacting the quality of life of SI-NET patients. As SI-NETs are relatively indolent tumours and OS continues to improve due to advances in treatment, the emphasis on quality of life becomes increasingly important.

Existing literature on MF is limited and challenging to compare due to variable diagnostic scoring systems and small sample sizes. Several promising initiatives aim to improve MF grading, including a standardized histopathological scoring system and measurement of five systemic PCR transcripts, which may facilitate better collaboration, comparability between studies and more clinically relevant correlations. A prognostic radiomics model is currently being validated, and other techniques – such as FAPI-based imaging – are emerging.

MF pathogenesis is another understudied area, affecting the understanding of the disease and the development of new therapies. While current literature heavily attributes the role of serotonin, *in vitro* research into its pathophysiological mechanisms remains sparse. General pro-fibrotic factors are known to be involved in MF; however, their interaction with disease-specific factors is not well described. While recent studies have made progress in exploring new disease pathways, giving focus to the tumour microenvironment, further investigations using patient samples and appropriate disease models are needed to uncover key mechanisms.

Recent advances in exploring new disease pathways present hypothetical therapeutic opportunities to inhibit MF progression in SI-NET patients – opportunities that are urgently needed. Currently, treatment remains limited to surgery, which is often complicated by extensive MF and frequently results in non-radical resections. While the development and testing of novel treatments for MF are still in their early stages, this emerging field holds great promise for improving patient outcomes and transforming disease management.

## Declaration of interest

MCM, ES, AB, HH, DM, CT, MEC and LJH have no conflicts of interest to declare. JH receives speaker or advisory board fees from Ipsen, Serb and Novartis. WWH has consultancy and advisory roles and receives honoraria from Ipsen, Novartis and Camurus and support for travel/accommodation from Recordati. RAF receives consultancy fees and research grants from Recordati and Corcept. KR owns shares in Engitix Therapeutics Ltd and receives consultancy fees from Engitix Therapeutics Ltd.

## Funding

This work was supported by the Neuroendocrine Tumor Research Foundation, NETRF Accelerator Grant 702627 (UCL/Erasmus).
